# A New Reversible Phase Transformation of Intermetallic Ti_3_Sn

**DOI:** 10.3390/ma12152484

**Published:** 2019-08-05

**Authors:** Minshu Du, Lishan Cui, Feng Liu

**Affiliations:** 1 School of Materials Science and Engineering, Northwestern Polytechnical University, Xi’an 710072, China; 2 State Key Laboratory of Heavy Oil Processing, China University of Petroleum, Beijing 102249, China

**Keywords:** Ti_3_Sn, phase transformation, crystal structure, X-ray diffraction, transmission electron microscopy

## Abstract

Ti_3_Sn has received increasing attention as a high damping metallic material and as an anode material for rechargeable lithium-ion batteries. However, a heated dispute concerning the existence of solid state phase transformation of stoichiometric Ti_3_Sn impedes its development. Here, thermal-induced reversible phase transformation of Ti_3_Sn is demonstrated to happen at around 300 K by the means of in-situ variable-temperature X-ray diffraction (XRD) of Ti_3_Sn powder, which is also visible for bulk Ti_3_Sn on the thermal expansion curve by a turning at 330 K. The new phase’s crystal structure of Ti_3_Sn is determined to be orthorhombic with a space group of Cmcm and the lattice parameters of a = 5.87 Å, b = 10.37 Å, c = 4.76 Å respectively, according to selected area electron diffraction patterns in transmission electron microscope (TEM) and XRD profiles. The hexagonal → orthorhombic phase transformation is calculated to be reasonable and consistent with thermodynamics theory. This work contributes to a growing knowledge of intermetallic Ti_3_Sn, which may provide fundamental insights into its damping mechanism.

## 1. Introduction

There are increasing attempts focusing on Sn-based anode materials for rechargeable lithium-ion batteries due to their high storage capacities [1,2,3,4], among which the intermetallic Ti_3_Sn seems to be the representative one [5,6]. Ti_3_Sn has received attention due to its ultra-high mechanical damping property in the wide frequency range of 1–200 Hz as well as 20–100 KHz [7], which is promising for energy dissipation applications such as vibration-resistant components and noise-cancelling products. The damping capacity Q^−1^ of Ti_3_Sn is almost one order of magnitude higher than that of commercial damping alloys including grey cast iron, Fe–Mn, Ni–Ti, Sn–Pb alloys, etc. [7,8]. The results of dynamic mechanical analysis (DMA) of Ti_3_Sn with a frequency of 1 Hz measured under heating reveal the interesting points: an obvious damping peak exists at about 310 K which just corresponds to the turning point of storage modulus; when the temperature is lower than 310 K, the damping capacity increases continuously [7]. Though the basic damping mechanism of Ti_3_Sn remains unknown presently, these experimental data suggest the appearance of a phase transformation in Ti_3_Sn at near-room temperature (~310 K).

According to the Ti–Sn equilibrium diagram [9], Ti_3_Sn melts congruently at 1943 K, and it remains stable down to room temperature without undergoing any phase transformations. The intermetallics has the ordered hexagonal D0_19_ crystal structure with a space group of P6_3_/mmc (No. 194) and the lattice parameters of a = 5.916 ± 0.004 Å and c = 4.764 ± 0.004 Å [10]. Colin McCullough et al. in 1993 studied the phase selection in undercooled Ti_3_Sn melts, and reported two previously unknown metastable forms of Ti_3_Sn: base-centered orthorhombic structure (space group: C_mmm_) with the lattice parameters of a = 9.76 Å, b = 6.18 Å and c = 4.75 Å in the sample with supercoolings of 109 K and 311 K; monoclinic structure with the lattice parameters of a = 5.72 Å, b = 4.74 Å, c = 6.05 Å and β = 79.5° in the sample with supercoolings of 258 K [11]. Ivanova, et al. has reported a reversible group-subgroup transformation between D0_19_ phase and new orthorhombic phase of non-stoichiometric Ti_3_Sn (Ti_75.5_Sn_24.5_) [12]. However, no new equilibrium structure of stoichiometric Ti_3_Sn has been reported. Even though the existence of equilibrium phase transformation of Ti_3_Sn has been discovered indirectly from its DMA results in [7], there are no direct experimental data about it, and the characteristics of transformation and crystal structure of new phase still remain unknown.

In this paper, by the means of thermal expansion measurement and in-situ X-ray diffraction during cooling/heating, thermal-induced reversible transformation of Ti_3_Sn was observed directly, and the crystal structure of the new phase was determined based on a set of electron diffraction patterns in TEM and XRD profiles. The results could provide fundamental insights into the properties of Ti_3_Sn in the application as the anode material of lithium-ion battery, since volume change and phase transformation during lithiation and delithiation process at different temperatures should be carefully considered, which may lead to the low storage capacity, poor cycling and even the cracking and crumbing of the metal electrode [13,14]. In addition, it is helpful to decode the mechanical damping mechanism of Ti_3_Sn and improve the development of Ti_3_Sn as a new generation of high damping metallic materials.

## 2. Experimental Section

### 2.1. Preparation of Samples

High-purity stoichiometric Ti_3_Sn intermetallic ingot with a nominal composition of Ti_75_Sn_25_ (atomic percent) was prepared by arc melting in a water-cooled copper crucible using 99.99% (atomic percent) purity starting elements. The ingot was melted eight times repeatedly and then homogenized under vacuum in a furnace at 1223 K for 10 h. There were two kinds of specimens used in our experiments: the bulk Ti_3_Sn samples were taken from the ingot with proper size by electric discharge machining, and the Ti_3_Sn ultrafine powders (with particle size of ~50 µm) which were obtained by grinding the bulk material in an agate mortar for 30 min and annealing at 673 K for 20 min under vacuum in a furnace in order to eliminate the residual stress that generated during the milling process.

### 2.2. DSC and Thermal Expansion Measurement

Differential scanning calorimetry (DSC) test was carried out on the NETZSCH 204 F1 using bulk sample in the Al_2_O_3_ crucible, and the heating/cooling rate was 10 °C/min in the argon atmosphere. Thermal expansion measurement was carried out on the WRP-1 equipment with a heating/cooling rate of 10 °C/min. The sample size for thermal expansion test was 25 mm × 2 mm × 1 mm according to ASTM (American society for testing and materials) standards. 

### 2.3. Transmission Electron Microscopy (TEM) Characterization

The sample for transmission electron microscopy (TEM) characterization was prepared by mechanical grinding, dimpling and ion milling, successively. TEM observations were carried out with a JEM-2100 microscope (JEOL, Tokyo, Japan) operated at a voltage of 200 kV. A Gatan K2 direct detection CCD camera (Gatan, Pleasanton, CA, USA) was used for image acquisition. It should be noted that the grain size of the as-cast Ti_3_Sn sample was in the range of hundreds of micrometers, and therefore the specimens fabricated for TEM observation were most likely to be from a single grain. The crystal structure of a new phase of Ti_3_Sn was determined based on a series of selected area electron diffraction (SAED) patterns. The reflection conditions were summarized according to SAED, which was then indexed to new phase structure parameters. Space group could also be uniquely determined based on reflection conditions.

### 2.4. XRD Analysis

In-situ X-ray diffraction (XRD) measurements during cooling process (320 K → 93 K) and heating process (93 K → 320 K) were carried out on a Bruker AXS D8 equipment (Bruker, Karlsruhe, Germany) using Cu K_α_ radiation with a wavelength of 1.54056 Å under a scanning rate of 2°/min. Ni-plated copper sample holder was fixed on the cooling or heating block. The cooling block was cooled by a continuous liquid nitrogen flow, and the heating block contained a heating strip made of AlCr. The new phase’s crystal structure was also solved using JADE (6.5 version, Livermore, CA, United States) and Jana software (2006 version, Praha, Czech Republic).

### 2.5. First Principle Calculation

The formation enthalpy of Ti_3_Sn was calculated using density functional theory (DFT) implemented in the Vienna Ab initio Simulation Package (VASP) with standard potential function [15,16,17]. All the structures were fully relaxed with respect to volume and the atomic coordinates. A cutoff of 360 eV was used for all elements. The settings of k-points correspond roughly to a 5000 k-point mesh per reciprocal atom. The formation enthalpy (with thermal contributions and zero-vibration energy difference or contribution being ignored) for an equilibrium phase Ti_x_Sn_y_ can be defined as [18,19]:

ΔH (Ti_x_Sn_y_) = E(Ti_x_Sn_y_) − x_Ti_·E(Ti) − y_Sn_·E(Sn) (1)

where E(Ti_x_Sn_y_) is the total energy of Ti_x_Sn_y_ phase per atom, obtained by first-principle calculations. E(Ti) and E(Sn) are the energy for the pure elements Ti and Sn, respectively. x_Ti_ = x\(x + y) and y_Sn_ = y\(x + y) are the fractional compositions of Ti and Sn, respectively.

## 3. Results and Discussion

### 3.1. Detection and Characterization of Phase Transformation of Ti_3_Sn

**Figure 1:**
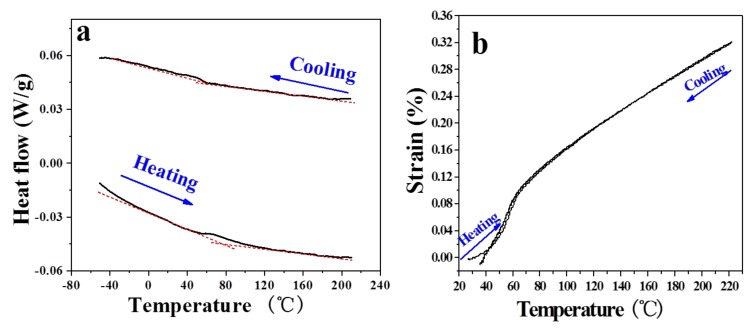
Phase transformation characterization of intermetallic Ti_3_Sn. (**a**) DSC result. (**b**) Thermal expansion test result.

At the beginning, conventional methods of DSC and thermal expansion tests were used to detect the phase transformation of Ti_3_Sn. DSC result in Figure 1a shows a wide and weak endothermic peak at about 330 K during heating process, and the corresponding exothermic peak during cooling process was too weak to be clearly observed. The thermal expansion measurement result in Figure 1b shows an obvious turning at about 330 K, which means the thermal expansion coefficient of Ti_3_Sn changes at this temperature. It is quite possible that the solid state phase transformation of intermetallic Ti_3_Sn indeed exhibits at room temperature (~330 K from thermal expansion test result) as suggested by DMA results in [7].

**Figure 2:**
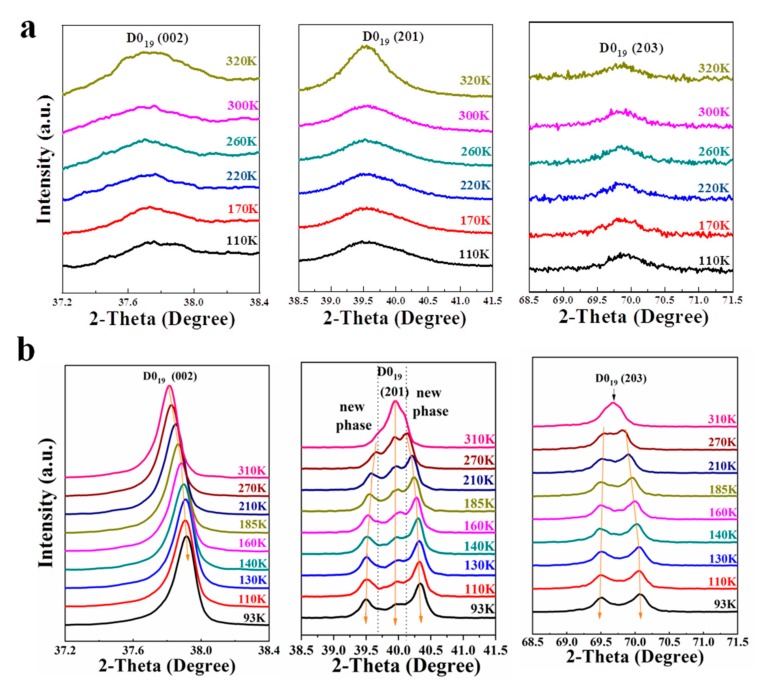
Comparison of conventional X-ray diffraction (XRD) patterns between (**a**) bulk Ti_3_Sn sample and (**b**) Ti_3_Sn powder during the cooling process. (**a**) XRD peaks of bulk Ti_3_Sn near hexagonal D0_19_ phase (002), (201) and (203) crystal faces. (**b**) XRD peaks of Ti_3_Sn powder near hexagonal D0_19_ phase (002), (201) and (203) crystal faces, respectively.

**Figure 3:**
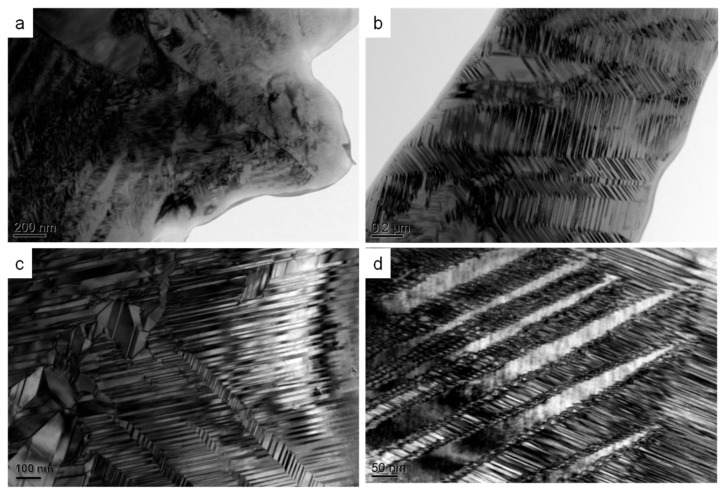
Overlapping twins in bulk Ti_3_Sn sample. (**a**) Extensively distributed twins; (**b**) lamellar nano-twins; (**c**) multiple twins distributed with 120° crossover; (**d**) stepped twins and inner lamellar twins.

In order to characterize the phase transformation directly, in-situ XRD experiments of both bulk Ti_3_Sn sample and Ti_3_Sn ultrafine powder were carried out during cooling process. It is true that the crystal structure of Ti_3_Sn at 320 K is ordered as a hexagonal D0_19_ structure with the lattice parameters as shown in [10]. However, entirely different XRD patterns of two kinds of Ti_3_Sn samples have been obtained during cooling process from 320 K down to 93 K. For bulk Ti_3_Sn, Figure 2a reveals no shape changes of hexagonal D0_19_ structural diffraction peaks (002), (201), and (203), and the peak positions are almost the same. While for Ti_3_Sn powder, Figure 2b reveals that D0_19_ (002) peak at about 37.8° at 320 K moves toward high diffraction angle direction gradually during cooling and changes to be at about 37.9° at 93 K; D0_19_ (201) peak around 39.9° at 320 K splits to two new peaks at 39.5° and 40.3° at 93 K; D0_19_ (203) peak at 69.6° at 320 K also splits into two new peaks at 69.4° and 70.1° respectively at 93 K during cooling process. It is known that diffraction peaks splitting and new peaks appearing are the proofs of symmetry-breaking phase transformation. As a result, we present the direct evidence of solid state phase transformation of Ti_3_Sn. By comparison, the XRD peaks of bulk Ti_3_Sn sample (with grain size of about 400 µm) are quite wider than that of Ti_3_Sn ultrafine powders (with particle size of ~50 µm as measured by SEM). The abnormal broadening of XRD peaks of bulk Ti_3_Sn was not fully understood, and it was speculated to be mainly caused by strain/stress generated from the overlapping nano-twins [20]. As shown in Figure 3, there are a mass of nano-twins in bulk Ti_3_Sn in the form of stepped twins, lamellar twins and polygon twins, the overlap and intersection of which would probably cause large strain or stress within the grain and result in XRD peak broadening. In addition, it is noted that the D0_19_ (201) peak existed during the cooling process till 93 K (shown in Figure 2b, middle subfigure), the area of which decreased gradually along with the area increases of two new phase’s peaks during cooling. Thus, it is indicated that phase transformation of Ti_3_Sn kept going during 310–93 K cooling process and did not complete leaving with a certain amount of hexagonal parent phase at 93 K. Such on-going phase transformation during cooling might be related to the continuously high damping (Q^−1^ ≈ 0.2) of Ti_3_Sn below 300 K, as shown in Figure 4a in [7].

**Figure 4:**
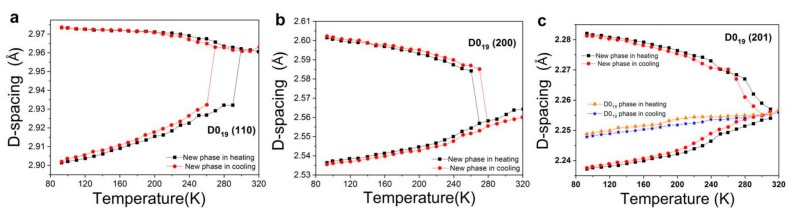
Lattice d-spacing variations of Ti_3_Sn powder during in-situ cooling and heating process. (**a**) D-spacing change of D0_19_-(110) plane; (**b**) d-spacing change of D0_19_-(200) plane; (**c**) d-spacing change of D0_19_-(201) plane in a temperature range of 93–320 K, respectively. The D0_19_ (201) peak still existed after phase transformation to the lowest temperature of 93 K.

Lattice d-spacing changes of (110), (200) and (201) peaks of powder Ti_3_Sn hexagonal D0_19_ phase during in-situ cooling and heating processes were indicated in Figure 4 respectively, in which the d-spacing value was calculated according to Bragg equation of: *2d* × sinθ *= nλ* on the basis of a set of Ti_3_Sn powder XRD profiles. From Figure 4, D0_19_ (110), (200) and (201) planes show obvious peak splitting into two new peaks during cooling from 320 K to about 93 K, while two new peaks also merge to the original D0_19_ peak during the following heating process. Thus, this symmetry-breaking phase transformation of Ti_3_Sn is proved to be reversible with a hysteresis of 10–30 K. For example, as shown in Figure 4a, lattice d-space of D0_19_ (110) at the temperature higher than room temperature (~300 K) is about 2.96 Å and splits into two new peaks at 270 K, d-spacing of one new peak increases and d-spacing of the other peak decreases as the temperature further goes down. It is noted that d-spacing increases during cooling indicating a negative thermal expansion (NET) of Ti_3_Sn in a certain temperature range. NET is generally known and occurs in zeolites, metal oxides, Aluminum phosphates, and so on [21,22]. The origin and mechanism for NET in Ti_3_Sn is currently unknown and needs to be studied in the future.

### 3.2. Crystal Structure of a New Phase of Ti_3_Sn

**Figure 5:**
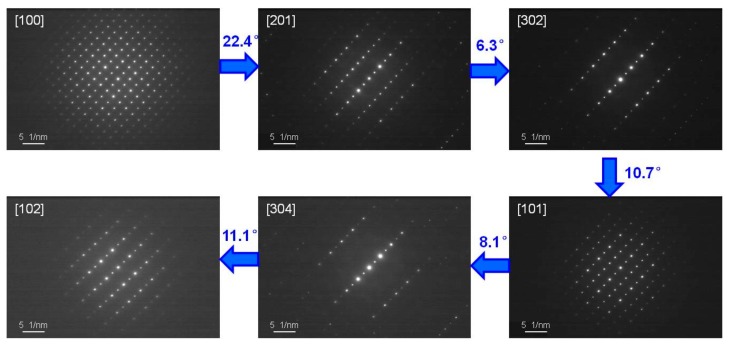
A set of selected area electron diffraction (SAED) patterns of Ti_3_Sn at the room temperature. The crystal zone axis and experimental tilting angle between the neighboring SAED patterns are indicated in the figure.

Two methods were employed to solve the crystal structure of the new phase of Ti_3_Sn. The first method for determining the crystal system and space group was based on SAED patterns of TEM. For Ti_3_Sn bulk sample, the phase transformation started at ~310 K from the previous DMA result [7] or ~330 K from our thermal expansion measurement result. Thus we can determine the new phase’s crystal structure of Ti_3_Sn foil specimen at room temperature (~290 K) by the traditional selected area electron diffraction technique of TEM. By systematically tilting the Ti_3_Sn specimen, a set of SAED patterns were obtained as shown in Figure 5. The crystal zone axis and experimental tilting angle between the neighboring SAED patterns are noted in the figure. Analyzing the patterns, the reflection conditions could be obtained as: for hkl, h + k = 2n; for 0kl, k = 2n; for h0l, h, l = 2n; for hk0, h + k = 2n; h00, h = 2n; for 0k0, k = 2n; for 00l, l = 2n. Thus, the reflections can be indexed according to a base-centered orthorhombic structure with lattice parameters of a = 6.10 Å, b = 10.45 Å, c = 4.89 Å, and the space group was uniquely determined to be Cmcm (No. 63) according to the International Tables for Crystallography [23].

**Figure 6:**
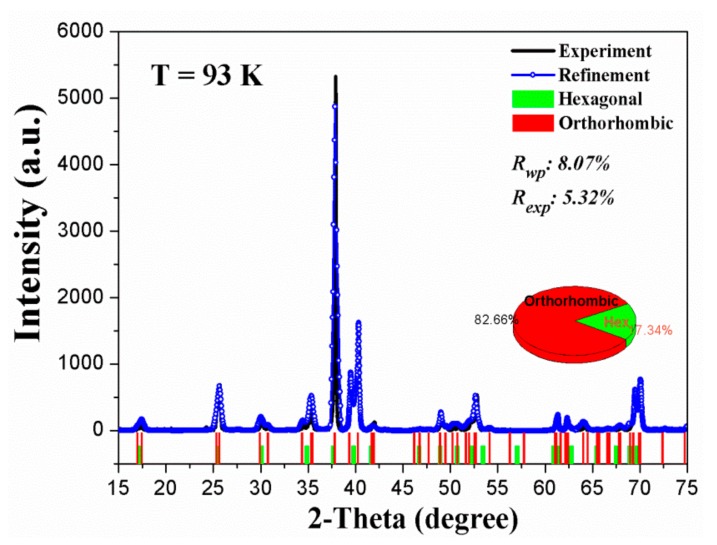
Rietveld refinement result of XRD profile of Ti_3_Sn powder at 93 K, in which the hexagonal phase and the orthorhombic phase positions were indicated by green bars and red bars, respectively. The phase contents were also shown by the pie sub-figure as inserted.

It is known that the accuracies of the measured d-values and the angles between the reflections of SAED are inferior to the accuracy in X-ray diffraction (XRD) due to the presence of electromagnetic lenses in the TEM [24]. Thus, crystal structure of the new phase of Ti_3_Sn was further determined from XRD profiles of Ti_3_Sn powder (shown in Figure 2b) using JADE and Jana software after Rietveld full-profile refinement. The refinement result of XRD profile of Ti_3_Sn powder at 93 K was shown in Figure 6, in which the hexagonal phase and the orthorhombic phase positions were indicated by green bars and red bars, respectively. Phase content could also be obtained, which turned out the existence of 17.34% parent phase (hexagonal phase) left without transformation at 93 K. XRD profile at 93 K was indexed to be base-centered orthorhombic structure with lattice parameters of a = 5.87 Å, b = 10.37 Å, c = 4.76 Å, and the space group was Cmcm, atomic Wyckoff positions were Ti (8g): (0.231, 0.904, 0.250), Ti (4c): (0, 0.636, 0.250), Sn (4c): (0, 0.163, 0.250), as listed in Table 1. Comparing the orthorhombic lattice parameters of a, b and c obtained from SAED and XRD, the differences were reasonable and within 5% measurement error of SAED. 

**Figure 7:**
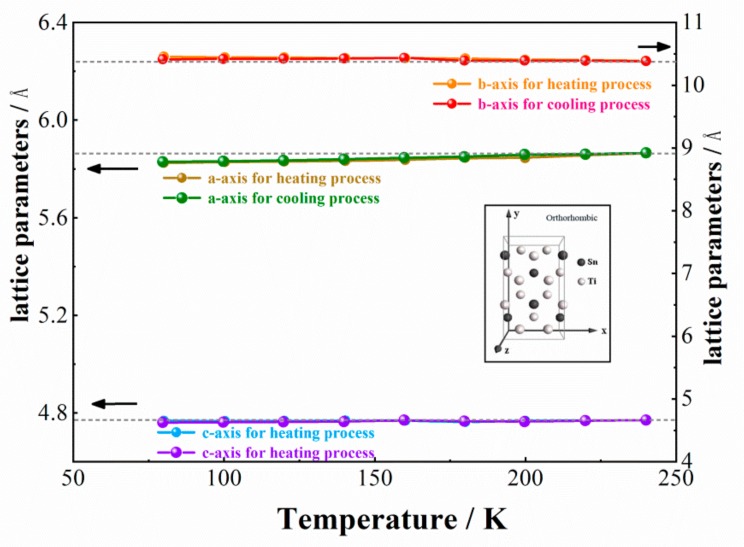
Temperature dependence of lattice parameters a, b and c of orthorhombic Ti_3_Sn during cooling and heating processes, in which the schematic unit cell is also showed.

Temperature-dependent lattice parameters of a, b and c for orthorhombic Ti_3_Sn obtained from XRD profiles were shown in Figure 7. It could be seen that parameter a reduced during cooling, parameter c remains almost constant, and parameter b increased during cooling. The d-spacing of (hkl) plane in orthorhombic phase is calculated according to Equation (2):

(2) $$d_{\mathrm{hkl}}\;=\;\frac{1}{\sqrt{{\left(\frac{h}{a}\right)}^{2}\;+\;{\left(\frac{k}{b}\right)}^{2}\;+\;{\left(\frac{l}{c}\right)}^{2}}}$$

Thus, when D0_19_ plane (201) was divided into the doublet of orthorhombic (041) and (221) planes during cooling as indicated in Figure 2b, d-spacing of (041) should increase as lattice parameter b increases and c keeps constant, and d-spacing of (221) should decrease as the reduction of a is larger than the increment of b during cooling, which are consistent with the results shown in Figure 4c.

**Figure 8:**
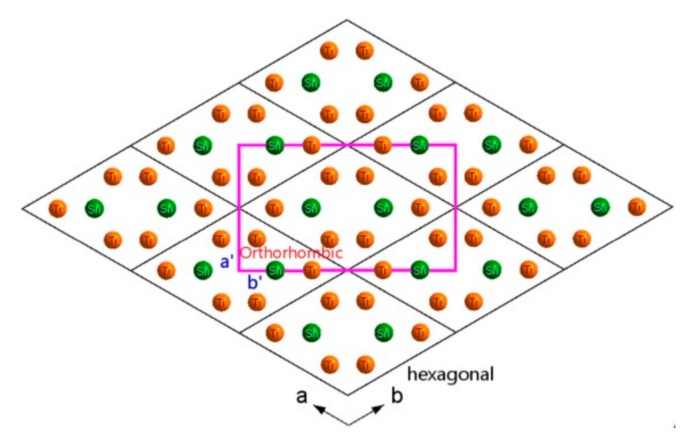
Comparison of the projection drawings of the hexagonal structure (drawn in black) and the orthorhombic structure (drawn in pink) of Ti_3_Sn from c axis, in which a, b axes of the hexagonal structure and a’, b’ axes of the orthorhombic structure were also indicated.

Furthermore, comparing the projection drawings of hexagonal structure and orthorhombic structure of Ti_3_Sn from the *c* axis as shown in Figure 8, the orthorhombic phase drawn in pink could be viewed as a distorted hexagonal phase with the main distortion in the ab plane since a_orthorhombic_ ≈ a_hexagonal_, b_orthorhombic_ ≈ √3b_hexagonal_, c_orthorhombic_ ≈ c_hexagonal_. Thus, the orientation relations between hexagonal parent phase and orthorhombic phase of Ti_3_Sn could be suggested as: (110)_orthorhombic_//(100)_hexagonal_, <001>_orthorhombic_//<001>_hexagonal_.

### 3.3. Discussion on the Thermodynamic Possibility of Phase Transformation

Formation enthalpies of both hexagonal phase (Ti_6_Sn_2_) and orthorhombic phase (Ti_12_Sn_4_) of Ti_3_Sn were calculated using Equation (2). Considering the sole outer electrons of atom, for hexagonal Ti_3_Sn (Ti_6_Sn_2_) on the basis of E(Ti_6_Sn_2_) = −7.073 eV, E(Ti) = −7.763 eV, E (Sn) = −3.835 eV, the formation enthalpy of hexagonal phase ΔH (Ti_6_Sn_2_) is −28.182 KJ mol^−1^ atom^−1^; for orthorhombic Ti_3_Sn (Ti_12_Sn_4_) on the basis of E(Ti_12_Sn_4_) = −7.078 eV, E(Ti) = −7.763 eV, E(Sn) = −3.835 eV, the formation enthalpy of orthorhombic phase ΔH (Ti_12_Sn_4_) is −28.654 KJ mol^−1^ atom^−1^. Also, when considering all the electrons of atom, E(Ti_6_Sn_2_) = −7.219 eV, E(Ti_12_Sn_4_) = −7.223 eV, E(Ti) = −7.891 eV, E(Sn)= −4.008 eV, so the ΔH (Ti_6_Sn_2_) is −28.848 KJ mol^−1^ atom^−1^, ΔH (Ti_12_Sn_4_) is −29.170 KJ mol^−1^ atom^−1^. Therefore, according to the results of the two kinds of calculations above, both hexagonal phase and orthorhombic phase of Ti_3_Sn are stable phases, and ΔH (Ti_12_Sn_4_) is more negative than ΔH (Ti_6_Sn_2_), which indicates that the orthorhombic phase is more stable than the hexagonal phase, and the phase transformation from hexagonal phase to orthorhombic phase is reasonable according to thermodynamics theory. 

## 4. Conclusions

The following conclusions can be drawn from this work:

- The reversible thermal induced phase transformation of Ti_3_Sn is demonstrated by the means of XRD and thermal expansion experiment;
- The temperature of forward phase transformation during cooling is 270–300 K, and this phase transformation is reversible with a hysteresis of 10–30 K according to XRD results;
- The phase transformation is determined to be a hexagonal → orthorhombic transition, the lattice parameters of the new orthorhombic phase are: a = 5.87 Å, b = 10.37 Å, c = 4.76 Å respectively, α = β = γ = 90°, the space group is Cmcm (No. 63). The atomic Wyckoff positions for orthorhombic Ti_3_Sn are also provided;
- The orientation relationships between orthorhombic and hexagonal phases are suggested as: (110)_orthorhombic_//(100)_hexagonal_, <001>_orthorhombic_//<001>_hexagonal_;
- The hexagonal → orthorhombic phase transformation is calculated to be reasonable according to thermodynamics theory.

These findings contribute to a growing knowledge of intermetallic Ti_3_Sn, and more comprehensive understanding of the formation mechanism of the orthorhombic phase is expected to be done in the future.

## Figures and Tables

**Table 1:** Crystallographic details of the new phase of Ti_3_Sn.

Phase: Orthorhombic		
Space Group: Cmcm (No. 63). 16 Atoms Per Unit Cell		
Lattice Parameters	Atomic Wyckoff Positions	
a = 5.87 Å	Ti (8g) Ti (4c) Sn (4c)	x: 0.231 y: 0.904 z: 0.250 x: 0 y: 0.636 z: 0.250 x: 0 y: 0.163 z: 0.250
b = 10.37 Å		
c = 4.76 Å		
α = β = γ = 90°		
